# Co‐designing and building an expert‐elicited non‐parametric Bayesian network model: demonstrating a methodology using a *Bonamia Ostreae* spread risk case study

**DOI:** 10.1111/risa.13904

**Published:** 2022-02-20

**Authors:** Anca M. Hanea, Zoë Hilton, Ben Knight, Andrew P. Robinson

**Affiliations:** ^1^ Centre of Excellence for Biosecurity Risk Analysis University of Melbourne Parkville Victoria Australia; ^2^ Cawthron Institute Nelson New Zealand

**Keywords:** Bayesian networks, expert elicitation, pathogens risk, uncertainty quantification

## Abstract

The development and use of probabilistic models, particularly Bayesian networks (BN), to support risk‐based decision making is well established. Striking an efficient balance between satisfying model complexity and ease of development requires continuous compromise. Codesign, wherein the structural content of the model is developed hand‐in‐hand with the experts who will be accountable for the parameter estimates, shows promise, as do so‐called nonparametric Bayesian networks (NPBNs), which provide a light‐touch approach to capturing complex relationships among nodes. We describe and demonstrate the process of codesigning, building, quantifying, and validating an NPBN model for emerging risks and the consequences of potential management decisions using structured expert judgment (SEJ). We develop a case study of the local spread of a marine pathogen, namely, *Bonamia ostreae*. The BN was developed through a series of semistructured workshops that incorporated extensive feedback from many experts. The model was then quantified with a combination of field and expert‐elicited data. The IDEA protocol for SEJ was used in its hybrid (remote and face‐to‐face) form to elicit information about more than 100 parameters. This article focuses on the modeling and quantification process, the methodological challenges, and the way these were addressed.

## 1 INTRODUCTION

In a general framework for risk assessment and management, the goal is to describe an uncertain system in which a hazard causes harm. For the risk assessment, four steps are necessary: hazard identification, hazard characterization, exposure assessment, and risk characterization (e.g., https://www.efsa.europa.eu/en/interactive_pages/riskassessment/RiskAssessment; EFSA, 2018). When the hazard is a pathogen, a cause‐and‐effect conceptual model from the prevalence of the pathogens to the probability and extent of harmful effects is often helpful to set the scene. Risk is commonly described as comprising both the probability and impact of harmful effects (in this case disease). Risk management and risk reduction can therefore be tackled in either or both dimensions, that is, controlling for the probability of disease and/or reducing disease severity. Understanding the available risk management decisions and their consequences is an integral part of decision making. A decision framework should allow groups of stakeholders to evaluate multiple‐objective decisions and analyze tradeoffs of various scenarios (sometimes called alternatives). However, formulating these alternatives and tradeoffs involves working with both facts (the uncertain state of the “world” one models) and values (what one deems important). Ideally the facts and the values should be kept separate when conceptualizing and parameterizing risk models. This distinction will help direct the search for, and use of appropriate resources.

To understand the facts, the values, and their interdependence, problem‐owners, analysts, stakeholders, and subject matter experts need to interact throughout the modeling process. These interactions may start as general discussions of their views, values, and uncertainties, and continue with more focused dialog about processes, specific inputs, outputs and associated uncertainties, available data, and expertise. A conceptual model is gradually constructed based on these discussions and guided by analysts. Building a consensus conceptual model (whenever possible) is crucial and it should happen before quantification is discussed.

Often the conceptual model remains underspecified, not all relevant stakeholders come to the table, or the process is not iterative enough for the stakeholders and the analyst team to reach consensus. Even more often, the subsequent steps are done without consulting a diverse enough group of experts, or without employing a structured approach when doing so.

The process of building and quantifying a risk and decision model with experts, stakeholders, and analysts is underpinned by methods and tools known in different disciplines by different names, e.g., problem structuring methods or knowledge engineering. Regardless of their name, most follow the same steps: They build a conceptual model, identify parameters, formulate data requirements, and address data gaps. Qualitative information flows back and forth along this as the model is shaped; it is an iterative process that oscillates between qualitative and quantitative elicitation. The boundary between qualitative and quantitative work is not as sharp as it may seem and this is driven by the fact that the distinction between parameters and model structure is, to some extent, shaped by the modeler's view and the available data. For example, in a simplified model, a parameter may represent the average value of the output of a submodel from a complex version of the same model. Similarly, sets of fixed conditions may be defined and formulated as scenarios to simplify the structure of the model, and hence make parameterization more tractable. The modeling process needs to then be treated as a whole. For a comprehensive discussion on this subject, see French (2021) and the references within.

A recent paper by Burgman et al. (2021) gives an overview of the available methods for model building and quantification, but argues that best practice advice cannot be proposed based on the existing literature. It however advises a participatory (and iterative) approach to model development that uses the most recent advances in structured expert elicitation techniques. This recommendation aligns perfectly with the method adopted in the current work. We refer the reader to Burgman et al. (2021) for a brief literature review of methods.

In this article we cover in detail the process of modeling the uncertain state of the world, and the way this model informs risk analysis. Conceptual and quantitative modeling is done collaboratively and iteratively, involving stakeholders and experts throughout the process. We focus on the process of modeling emerging risks when this collaborative approach is employed. Even though such approaches are invoked and indeed used often in risk analysis, few are comprehensively described and placed in the context of structured expert judgment (SEJ) for qualitative and quantitative modeling. We highlight the importance of semistructured and structured methods for eliciting, combining, and using expert judgments to build and quantify probabilistic models that are then ready to use in decision making. This process is described in the context of a risk and decision analysis regarding the spread of a fairly novel pathogen that could affect both the aquaculture industry and the wild fisheries of New Zealand. Even though the focus of the article is on the methodology, rather than the application, the latter will still be described in detail. There are many reasons for the detailed exposure: One is the need for enough clarity to enhance the coherence of the article as a stand‐alone piece, another is the importance of the application itself (since it is the first building block of a much larger modeling exercise). Lastly, the level of detail is not only indicative of potential complexities in any other application, but it also helps the reader to better understand the concrete methodological steps.

The remainder of this article is organized as follows: Section 2 describes the probabilistic modeling tools employed, the structured elicitation protocols proposed, and the measures used to verify and validate expert input. Section 3 provides detail about the *B. ostreae* case study and the resulting model. The article concludes with a discussion of advantages and limitations of the current work, and proposals for future work in Section 4.

## 2 PROPOSED METHODOLOGY

Modeling the uncertain state of the world often requires the assessment of multiple, dependent uncertain quantities of interest. In addition to univariate distributions, interdependencies between these quantities/variables need to be modeled, to properly understand the overall risk. Various probabilistic dependence models could be used to represent multivariate distributions. The most advantageous models provide a transparent and efficient way to model complex relationships, while being able to integrate data from different sources and with varying degrees of uncertainty.

Maybe the most popular such models and best supported by available (often free) software are Bayesian networks (BNs), for example, Pearl (1988). BNs are directed acyclic graphs, which provide a relatively simple visualization of potentially complicated relationships among the random variables. This is a huge asset when building collaborative conceptual models, because it enhances the experts' ability to construct and reason with causal and correlational models about problems from their area of expertise. Nevertheless, domain experts are not expected to construct their own BNs, but rather to contribute to conceptual models which can then be translated into BNs.

In BNs, the nodes of the graph correspond to random variables and the arcs represent direct qualitative dependence relationships. Arcs point from a parent node to a child node. The absence of arcs warrants a set of (conditional) independence facts. The nodes and arcs alone are called the structure of a BN, or the qualitative part of a BN. Marginal and conditional probabilistic distributions are specified for variables relating to each node, and they are called the quantitative part of a BN. The graph with the conditional independence statements encoded by it and the (conditional) distributions, together represent the joint distribution over the random variables denoted by the nodes of the graph. The quantitative information needed can be either retrieved from data, when available, or elicited from domain experts. Often a combination of the two is necessary.

Once fully quantified, BNs are used to update multivariate distributions with new information (observations or measurements) as it becomes available. This is referred to as inference in BNs. Another huge advantage of BNs is that they can be easily augmented with decision nodes representing, for example, management actions, and value nodes, representing costs associated with such actions.

BNs have been successfully used to represent uncertain knowledge, in a consistent probabilistic manner, in a variety of fields, for example, Pourret et al. (2008) and Weber et al. (2010). Ideally a large, complex BN is used to describe a complex problem. However, the number of parameters that require quantification (with data and/or expert judgments) increases with increased complexity. A careful balancing act is required to keep the model simple, yet realistic and useful.

What follows is a general description of the methodological tools proposed, together with details about the specific application. These descriptions are meant to ensure the tractability of the modeling process.

### 2.1 Methodological Tools

Most of the applications use discrete BNs (BNs whose nodes represent variables with discrete states). Despite their widespread use, discrete BNs are severely limited in case of applications involving potentially continuous and highly dependent quantities in data‐sparse settings. These limitations are mainly driven by the quantification process. When a variable depends on many others, this translates into a child node with many parents in a BN. The conditional distribution of such child node will then be represented as a conditional probability table with as many entries as the product of the number of discrete states of the child and its parents. This leads to an excessive assessment burden, which in turn may lead to informal and indefensible quantification, for example, Cooke et al. (2007), especially when data are scarce.

To avoid these problems, we chose a particular type of BN that can accommodate ordinal variables measured on both discrete and continuous scales, without restricting the continuous part to be described by a parametric family of multivariate distributions (e.g., a joint normal distribution). These are the hybrid nonparametric Bayesian networks (NPBNs), for example, Hanea et al. (2015). These models' parameterization, hence its quantification process (discussed in the next section), are relatively painless even in data‐sparse environments, where expert judgment is required. This is because it reduces the quantification burden and it circumvents most of the complications associated with eliciting and combining discretized conditional distributions from experts (see Charisse Farr et al., 2020, and references therein).

Using BNs (in general) facilitates the elicitation of the conceptual model, while using NPBNs facilitates the elicitation of the quantitative part of the model. Although the latter can be done in the context of SEJ, the former can only be semistructured. Eliciting a qualitative (conceptual) model should use natural language, so that experts feel comfortable when describing the processes involved. The elicited model should then have the potential of being translated into a probabilistic model. Even though BNs lend themselves to conceptual modeling in a natural and graphical way, sometimes, their logic does not match with the experts' description of a problem. Guidelines for how to overcome these situations are discussed in Kevin and Nicholson (2009), Smith (2010), and Wilkerson and Smith (2020).

#### 2.1.1 NPBNs

When quantifying BNs, univariate marginal distributions are required for the nodes without parents, and conditional distributions are required for the nodes with parents. Each child node is quantified through its conditional distribution given its parents in the graph. The NPBNs depart from the “classical” approach of quantification and instead require univariate marginal distributions for all the variables represented as nodes, and a dependence structure built on bivariate pieces of dependence, parameterized by rank correlations and conditional rank correlations associated with the arcs of the network. To build the multivariate dependence structure, (conditional) one‐parameter copulae, for example, Joe (1997) are used. The quantification of NPBNs reduces to the quantification of a number of marginal distributions equal to the number of variables and a number of (conditional) rank correlations equal to the number of arcs of the NPBN. These can be estimated from data or elicited from experts. Elicitation protocols are available, and have been successfully applied with domain experts in a number of applications (Hanea et al., 2015). For the mathematical details of the NPBNs, we refer to the original references, for example, Cooke et al. (2007) and Hanea et al. (2006).

After the NPBN is built and quantified, the multivariate joint distribution becomes attainable. Since a parametric form of the joint distributions is not necessarily available, the only way to stipulate it is by sampling it. General sampling procedure for NPBNs are detailed in Hanea et al. (2006). An analytic procedure becomes available when a particular type of copula is assumed (e.g., the normal copula).

Building the dependence structure from elicited correlations, or from separate bivariate data sets requires constructing a correlation matrix that must be semipositive definite. Optimization methods can be used to find the semipositive definite correlation matrix nearest to the one constructed from separate pieces of information. This is a neighboring matrix, which differs least from the input matrix, when the measure of nearness is expressed in terms of weighted norms, for example, Higham (2002).

#### 2.1.2 SEJ Protocols

Before discussing the technicalities of elicitation protocols, we will briefly introduce the actors involved in an elicitation. They can be grouped into an elicitation team and an expert group. The elicitation team often consists of decisionmakers (who identify the need of the analysis and contact the analysts), stakeholders, analysts, and modelers (who design the elicitation, contact facilitators, implement the resulting models, and analyze results), facilitators (who facilitate discussion and manage experts' interaction), and a couple of domain experts (who help in formulating the elicitation questions and advise on the elicitation format). The experts in the expert group are the ones whose judgments are elicited during an elicitation exercise. Often the same people can play multiple roles, but if they do, it is important for their roles to be clearly specified, and for motivational biases to be minimized.

The definition of an expert or of the required expertise in conceptual modeling or uncertainty quantification settings is still vague, but experience and common sense suggests that a diverse, large group of experts is the best choice. A discussion and relevant references on this topic are available in Hanea et al. (2021).

Structured protocols for eliciting expert judgments should subject these to the same level of empirical control and transparency as would be expected from data‐driven methods (Cooke, 1991; Drescher & Edwards, 2018; French, 2012). When these judgments are quantitative, the following elements were identified as necessary for an accountable, transparent, and repeatable, hence a *structured protocol*:

1. asks questions that have clear operational meanings, that is, something that in principle would be measurable if time and resources would permit experiments;
2. follows transparent methodological rules, that is, is traceable, repeatable, and available to review;
3. anticipates and mitigates some of the most important psychological and motivational biases^1^;
4. the process is thoroughly documented; and
5. provides opportunities for empirical evaluation and validation.^2^

A few of the requirements would be incredibly difficult to achieve when eliciting qualitative judgments, with the last one being simply impossible. The empirical control requirement provides an empirical basis for validating expert judgments when these are quantitative judgments. A further potential limitation of any protocols for eliciting qualitative judgments is that it has to strive for consensus, because qualitative judgments cannot be mathematically aggregated. For these reasons we qualify the elicitation described in this article for the BN's qualitative part as a semistructured elicitation, and the one for the quantitative part as a structured elicitation, where all five steps were taken.

In the context of NPBNs one needs to elicit marginal distributions and dependence parameters. Eliciting univariate (continuous) marginal distributions in a structured way has been applied for around 30 years (Cooke, 1991). Eliciting correlations, however, is fairly new in comparison and relevant references are Quigley et al. (2013) and Werner et al. (2017).

Univariate distributions are elicited by asking a finite number of percentiles (usually three). Often the 5% and the 95% percentiles are elicited together with the median. Correlations are best elicited directly as a measure of the strength of relationships between pairs of variables. The possible values of these correlations run from −1 to 1 where −1 corresponds to a completely negative relationship (the smallest values of a variable correspond to the largest values of the other variable) and +1 corresponds to a completely positive relationship (the smallest values of a variable correspond to the smallest values of the other variable) and 0 corresponds to no discernible monotonic relationship.

A variety of protocols for (quantitative) SEJ elicitation exist (EFSA, 2014; O'Hagan, 2019). In this project, we employed the IDEA protocol (Hanea et al., 2016). The acronym IDEA arises from the combination of the key features of the protocol that distinguish it from other structured elicitation procedures: It encourages experts to Investigate and estimate individual first round responses, Discuss, Estimate second round responses, following which judgments are combined using mathematical Aggregation. Previous research using this protocol suggested that, when using panels of experts, (facilitated) interaction improves the experts' ability to appropriately quantify uncertainty, and that performance‐based mathematical aggregation of expert judgments shows promise under circumstances involving external validation, for example, Hemming et al. (2020).

The performance‐based mathematical aggregation is an option only when performance can be measured. To measure performance, calibration questions are needed. Calibration questions are questions of which the true answers are known (with certainty) by the analyst or can be found within the time span of the study, and are not yet known by experts. They form a calibration data set used to gauge the quality of expert judgments. This quality is scored in terms of how calibrated and informative expert judgments are. The calibration and informativeness scores are then combined to form differential (performance) weights. Each expert's judgments can be weighted differently in a linear combination of all judgments, which will then serve as the group (aggregated) judgment. An equally weighted combination is used in absence of calibration questions.

#### 2.1.3 Validation and Verification

Building and quantifying models with experts in data‐poor environments result in models that are very hard to validate in a traditional statistical sense, since testing the model against an independent data set (not used in quantification) is impossible.

In the absence of data, different methods for verification and cross‐examination must be employed and tolerated, at least until new data are collected. Approaches to verification include face validity and case validation. Face validity is basically unstructured expert judgment, and case validation implies certain inferences whose outputs are checked against deterministic situations. For example, simple cases of inference using the proposed model are compared to known results from already performed experiments.

The other type of verification available is that of the quality of expert judgments in terms of calibration and informativeness. We use the calibration and informativeness measures defined by Cooke (1991). Calibration is measured as the *p*‐value at which the hypothesis that the expert is well calibrated would be falsely rejected. This score ranges from 0 to 1. Higher scores are better, since a low value (near 0) means that it is very unlikely that the discrepancy between an expert's probability judgments and the observed outcomes arose by chance. Informativeness is measured as the Kullback–Leibler divergence (Kullback & Leibler, 1951) with respect to the uniform distribution (which is what one would assume in absence of experimental or expert‐elicited data). Informativeness scores are larger than or equal to 0, with higher scores being better. For precise definitions and detailed analysis of these scores, we refer the reader to Hanea and Nane (2020).

A good probability assessor (or a good aggregated judgment) is one whose assessments capture the true values consistently in the long run (well calibrated), with distributions that are as narrow as possible (informative). Informativeness is gauged by “how far apart the percentiles are” relative to an appropriate background (e.g., the uniform distribution). Measuring calibration requires an analysis of the true values relative to the experts' assessments. When eliciting the 5%, 50%, and the 95% percentiles, an expert is considered well calibrated if, in the long run, 5% of their answers fall below the fifth percentile, 90% of the answers fall between the 5th percentile and the 95th percentile, and 5% of their answers fall above the 95th percentile. In gauging overall performance, calibration is more important than informativeness. Noninformative but calibrated assessments are useful, as they sensitize us to how large the uncertainties may be; highly informative but not calibrated assessments are worse than useless.

The performance of the experts on the calibration questions is taken as indicative for the performance on the target questions. Therefore, the calibration questions must resemble as much as possible the target questions. The more calibration variables the better, but 10 has proven to be sufficient, without adding too much to the elicitation burden, for example, Colson and Cooke (2017). A fundamental assumption of using calibration questions to measure performance is that the future performance of experts (as uncertainty assessors) can be judged on the basis of past performance, reflected by the calibration questions.

### 2.2 The Proposed Process

The process of modeling for this research followed a sequence of steps that we recommend as general guidelines. For more clarity we will describe many of these steps in the context of the present case study, but they are translatable to any other application area. A few of these steps and procedural choices, made when designing the elicitations (of the model and its parameters), are common to both the semistructured qualitative elicitation and the structured quantitative elicitation. They concern the elicitation team and the expert group compositions and sizes. In this exercise the problem owners acted as experts (from the expert group) in the quantitative elicitation and modelers in the qualitative conceptual modeling. A few stakeholders acted as experts (from the expert group) in the quantitative elicitation, but their judgments were on uncertainty quantification of facts (on which they were considered experts), rather than values, and hence no motivational bias was suspected. One of the facilitators was also responsible for modeling and analysis of results.

In this research we have contacted and involved 20 experts, even though the current recommendations are between 4 and 10,^3^ for example, Hanea and Nane (2020) and O'Hagan (2019). The reasons for deciding on a much larger group were to accommodate possible dropouts, to use a couple of experts advice when formulating the quantitative questions, and to achieve maximum diversity in terms of affiliation, interests, gender, seniority, and type of experience (e.g., modelers, observationalists, farmers). When all experts confirmed their participation we decided to use the entire group of experts for building a consensus conceptual model. In this way, we made sure that all expert's feedback was incorporated prior to engaging in a quantitative elicitation. This is essential, since the quantitative elicitation's needs are completely determined by the conceptual/qualitative model.

Modeling was initiated by the problem owner and brought forward to the modelers, for drafting an initial BN structure (see Figure A3). The initial BN structure was circulated to the group of 20 experts who were asked to scrutinize it and provide feedback. Each expert provided independent feedback to the modelers who collated, anonymized it, and incorporated it in the BN structure. Most feedback was consistent among experts, sometimes complementary, and easy to translate into changes to the BN structure. However a subgroup of four experts provided very critical feedback. The elicitation team decided to address this feedback in a face‐to‐face one‐day workshop. Even though this workshop was not planned for initially, it was deemed necessary in due course. This highlights the need for flexibility and fluidity of the process in the early stages. During this workshop the model structure was finalized, data needs (and potential data sources) were identified, and decisions about the type of expert judgment needed were taken. The final BN and the proposed plan for quantification were sent once more to all experts. No additional feedback was received.

Reconciling the feedback from all experts is a crucial modeling step, as the same experts need to be comfortable quantifying parts of the model they have carefully considered and scrutinized. Agreement on the assumptions of the model and the definitions of the variables ensures that all experts understand and answer the quantitative elicitation questions in the same way.

We initiated the quantitative elicitation process with sessions of remote presentations of the methods, of the reasons behind each step of the IDEA protocol, and of the format of the elicitation questions. During these sessions, practice questions were answered and discussed. Prior to receiving the questions for the SEJ elicitation, the experts received a plain language statement document, a consent form (to be signed), and an instructions document on how to answer the questions. The first round of estimates was completed remotely, and the answers were collated prior to a two‐day face‐to‐face workshop. Eighteen experts participated in the elicitation and answered more than 100 questions.^4^ The number of questions was determined by the quantification needs of the conceptual model (the qualitative part of the NPBN) and the data gaps identified by the expert group. The type of questions was determined by the NPBNs' quantification needs: univariate distributions for the nodes and rank correlations for the arcs.^5^ Univariate distributions were elicited by asking for a lower bound, an upper bound, and a best estimate for all quantities involved. We then considered the best estimate to correspond to the median of the subjective (personal) distribution assigned to the variable in question: the lower bound to correspond to the 5% percentile; and the upper bound to correspond to the 95% percentile of the subjective distribution. When eliciting correlations, experts were asked to provide upper and lower plausible bounds as well as best estimates for the strength of relationships between pairs of variables.

During the initial remote presentation, experts were briefly trained in quantifying their own uncertainty in terms of nominating three percentiles of their subjective distributions, and in quantifying rank correlations. Most of the experts were very familiar with these concepts.

The questions answered by experts prior to the SEJ workshop fall in two categories: calibration and target questions. The target questions are those about the parameters or the distributions for which experimental data are not available. For this project, there were 15 calibration questions constructed with the help of two international experts on *B. ostreae*. Given their involvement in formulating and answering the calibration questions, these two experts could not participate in the quantification workshop.

The quantitative elicitation protocol chosen for this project (the IDEA protocol) involves a face‐to‐face discussion stage (organized as a workshop) after an initial independent first round of estimates. The discussion is believed to be most efficient when using groups of less then 10 experts (for purely practical reasons). Because of this, we decided to split the group of 18 experts in two subgroups.

A two‐day face‐to‐face SEJ workshop (that took place in May 2019) started with a group discussion about the formulation of the questions in general, and about the fairness and appropriateness of the calibration questions. After this discussion the experts were distributed into two groups that aimed to mimic the diversity of the larger group, while keeping the group of experts small enough for the discussion to be optimal. Each group of experts had a facilitator who presented collated and anonymized answers to each question in turn, and facilitated discussion.

After discussing the reasons behind estimates and possible values for each variable, the experts had the chance to give a second, private/anonymous round of estimates. At the end of the elicitation session each variable had 18 different distributions (one per expert), which had to be aggregated for a final quantification. A linear combination of distributions per variable is maybe the most used mathematical aggregation technique. This linear combination can use equal or differential weights, with the latter being informed by experts' performance. For this exercise, both equal weighting and performance‐based weighting were evaluated. This evaluation was done for all possible different combinations, per group and for all experts (from both groups) pooled together. These combinations can be thought of as other experts, whose assessments incorporate all experts' assessments, and can be scored for performance using the same scores.

The approach for collecting and collating expert judgments ensures face validity. Moreover, after the model was fully quantified, several case validity tests were performed with satisfactory results. However, the most rigorous way to ensure the reliability of a model quantified with expert data is to make sure the expert data are well calibrated and the choice of the aggregation method is well justified. We used the calibration data set for both these purposes. We first evaluated the quality of the expert judgments and then we used those evaluations to create aggregated data. If the aggregations' performance is thought of as a goodness‐of‐fit measure, then the “best” aggregation is constructed such that they best fit the expert data, and they deliver the best performance.

All experts and experts' aggregations were evaluated in terms of calibration and informativeness. The best performance was achieved when all 18 experts' distributions were combined, with each distribution contributing an amount proportional to the experts' performance on 11 of the 15 calibration questions. The four excluded calibration questions were identified through a robustness analysis. Such analysis reveals questions poorly answered by all (or the majority of) experts. The rather subjective decision to exclude such questions is based on the assumption that those questions were either poorly formulated or misunderstood by the majority of experts, discarding them as fair questions.

The aggregation, selected as the best combination to represent the expert group, achieved a much higher calibration than any individual expert. Even though its informativeness score was only better than two of the individual informativeness scores, the overall performance was deemed the best, because the calibration drives the combined score. The aggregated “expert” representing the group achieved a calibration of 0.74. Informativeness scores larger than 3 are considered excellent. This group's informativeness score was 3.49. These scores are computed across questions, hence interval information is not available, however robustness analysis can be performed. In this case, robustness analysis is done by recalculating the scores that would have been obtained if questions were excluded one at a time. This exercise resulted in calibration scores ranging from 0.55 to 0.74, and informativeness scores ranging from 3.04 to 4.05.

Eliciting correlations and assessing the quality of such assessments is much more difficult and less studied (Werner et al., 2017). Good performance on the type of calibration question appropriate for quantifying continuous distributions did not predict good performance when correlations were elicited in the only study that studied this relationship (Morales‐Napoles et al., 2014) that the authors of this article are aware of. Therefore, in this research, because none of the calibration questions were about correlations, we assumed that the performance measures calculated said nothing about how well correlations may have been estimated by experts. As a consequence, the best estimates^6^ of the correlations were simply averaged.

Because the correlations forming a correlation matrix are constrained by the positive‐definitive requirement, after averaging we have calculated the closest positive definite matrix to the one obtained. This was the only theoretical verification performed for the correlation structure.

The aggregated distributions and correlations were sent to the experts for them to compare and contrast their personal estimates with the group estimates. There was no disagreement about these results.

**FIGURE 1:**
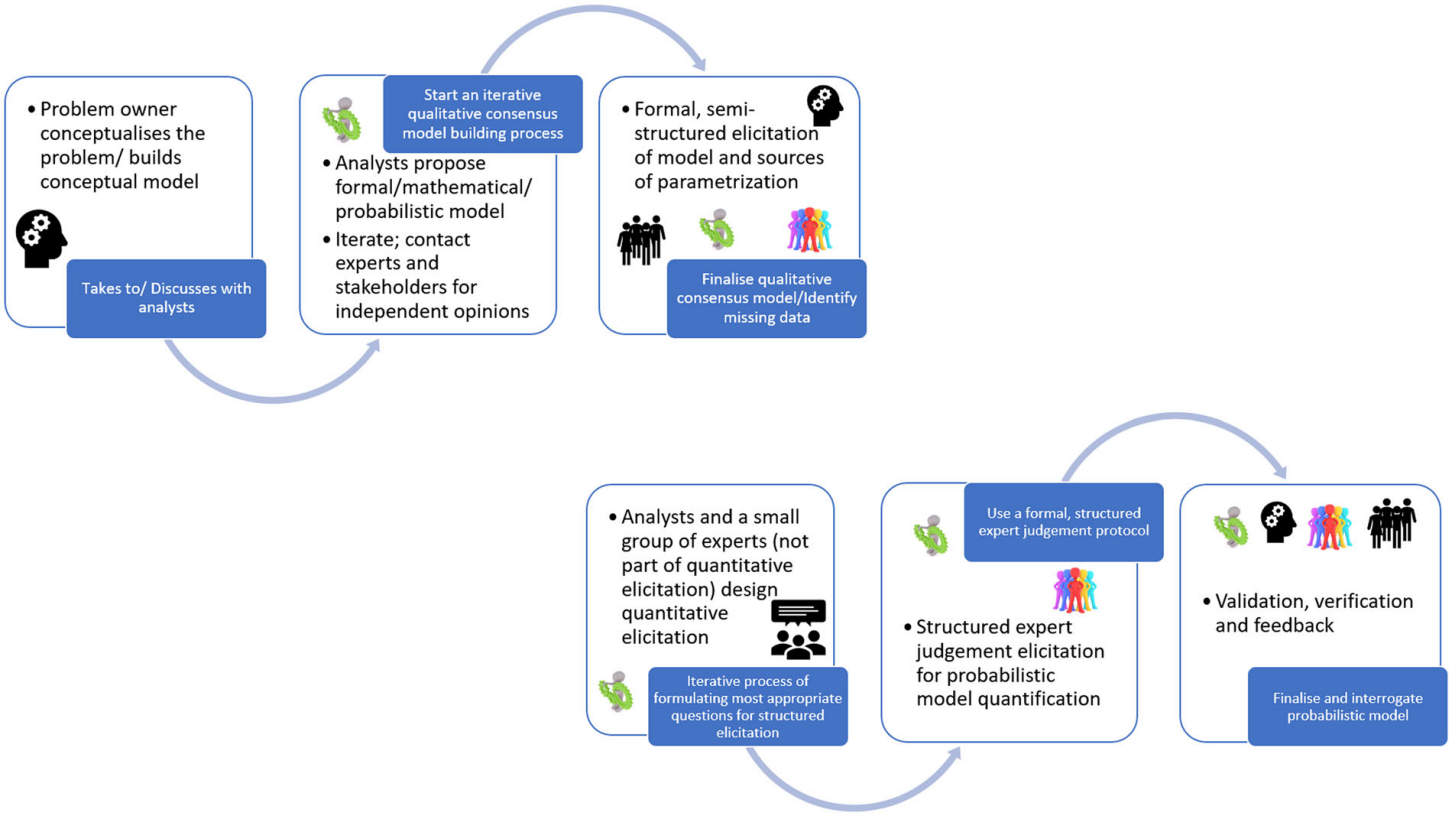
Schematic representation of the proposed process. The two parts of the process correspond to the qualitative semistructured elicitation (top) and the quantitative structured elicitation (bottom)

A schematic representation of the proposed process is depicted in Figure 1. The top part of the figure corresponds to the semistructured elicitation of the qualitative part of the model. The lower part represents the steps involved in a quantitative SEJ. Even though the two parts seem disconnected, in practice they can rarely be, as the needs of one informs the other. The choice to depict them as such is meant to emphasize that the qualitative modeling should be finished prior to engaging in the quantitative part of the process.

## 3 CASE STUDY

The rest of this article is dedicated to the case study of *B. ostreae* in the New Zealand oyster *Ostrea chilensis* (*O. chilensis*). Even though the focus of this article is on the methodology, the presentation of the case study will include enough details for the article to be (1) a stand‐alone piece (from the application perspective) and (2) a useful applied guide to probabilistic modeling aided by expert elicitation.

Due to its remote location, New Zealand's marine environment is free of many pathogens and invasive species that are common elsewhere. However, a variety of pathways for the arrival of new pathogenic species exist (e.g., hull‐fouling or ballast water discharge from international shipping and recreational boating, importation of ornamental species and bait products). It is globally recognized that managing shellfish health and production requires considerable risk management (Carnegie et al., 2016), and in the last decade, at least two pathogens (OsHV‐1 μ var and *Bonamia ostreae* [*B. ostreae*]) have had major impacts on New Zealand's shellfish aquaculture industries. We chose one of these pathogens, namely, *B. ostreae*, as a case study. *B. ostreae*, a parasite which infects flat oysters, was previously thought to be present only in the northern hemisphere, and was first detected in New Zealand in 2015, in the Marlborough Sounds (Lane et al., 2016). Its appearance was associated with unusually high levels of mortality in high‐density growing systems on farms (A. Elliot and B. Hearn, personal comm, 2019). According to retrospective testing of available archived samples, the parasite was not detected prior to 2014, and it is thus believed to be a recent introduction to New Zealand (Lane, 2018). *B. ostreae* is thus currently classified as both a “New Organism” and an “Unwanted Organism” under the 1996 New Zealand Hazardous Substances and New Organisms (HSNO) Act. In 2017, *B. ostreae* was detected via targeted surveillance in farms in Stewart Island's Big Glory Bay. This location is directly adjacent to the iconic wild flat oyster dredge fishery in Foveaux Strait in southern New Zealand, hence this detection was of great concern. Subsequently, the government decided to close all flat oyster aquaculture operations in both locations (Marlborough and Big Glory Bay), due to concerns that actual or potentially infected stocks posed an unacceptable level of risk of spread of *B. ostreae* to the wild fishery. Farmed animals were removed from all farms except one that was located on Stewart Island outside of Big Glory Bay (considered to be a separate epidemiological unit), where *B. ostreae* was not detected.

To date, no *B. ostreae* has been detected in the surrounding wild populations of Foveaux Strait, or Tasman and Cloudy Bays (NIWA, 2018), but infection has been found in other wild populations in the Marlborough Sounds. In October 2019, one wild oyster from Big Glory Bay has tested positive for *B. ostreae* (through the https://www.biosecurity.govt.nz/news‐and‐resources/media‐releases/single‐wild‐oyster‐infected‐with‐bonamia‐ostraeae‐found‐in‐big‐glory‐bay/?utm_source=notification‐email, Biosecurity New Zealand's surveillance programme). In March 2021, three oysters from Foveaux Strait were reported as infected, but these reports were (https://www.mpi.govt.nz/news/media‐releases/bonamia‐testing‐process‐for‐oysters‐strengthened/?utm_source=notification‐email) declared faulty later in the year by the Ministry for Primary Industries.

Future management actions and surveillance efforts can be better informed by a more thorough risk (of spread) analysis. Such analysis was initiated in 2018 by researchers at Cawthron Institute, under the Programme: Aquaculture Health to Maximise Productivity and Security. Interventions such as total or partial removal of diseased/moribund animals, or stricter rules and regulations for oyster farming may help control the density of oyster populations and the potential infection prevalence in such already infected populations. However, the effects of such interventions on the likelihood of a disease outbreak remain uncertain. We aim to model this uncertainty using a multivariate model that incorporates as many interdependent variables as possible. We restrict the modeling to its probabilistic aspects, excluding the cost and benefits of management actions for the moment. However, we argue that their inclusion would be straightforward and would allow for a complete quantification of risk in terms of both the probability of *B. ostreae* spread, and its impacts.

This case study is the first step toward a more complex risk analysis of spread of *B. ostreae* from a local scale, to regional and national scales. Here, we try to model the experts' understanding of the characteristics of a potential outbreak at a small scale, under simplifying conditions. However, thanks to the flexibility of the probabilistic models chosen for this application, we are confident that the model can be improved (when more data become available), scaled, and integrated into a larger body of work (including hydrodynamic models, regional and national network models). As it stands, the model is more a proof of concept than a final evaluation of the spread risk.

This section starts with a brief description of the environmental background and continues with a description of the modeled processes and of the way the existing literature and expert advice shaped the conceptual modeling. Section 3.2, even though not intended as a formal systematic literature review, does cover most of the available references relevant to this case study. The detailed description of the constructed and quantified local NPBN concludes this section. The exact quantification of this model is not the focus of the article, but the joint distribution of the variables will be discussed and (aspects of it) scrutinized.

### 3.1 Background

Parasites of the genus Bonamia have had severe impacts on farmed and wild populations of flat oysters around the world (Bradley et al., 2020; Carnegie et al., 2016; Doonan et al., 1994; Engelsma et al., 2014), hence two of the species, namely, *B. ostreae* and *Bonamia exitiosa* (hereafter *B. exitiosa*), are notifiable organisms to the World Organisation of Animal Health OIE (2018a, 2018b). Many flat‐oyster producing countries have national and regional control measures in place to prevent the spread of Bonamia, for example, Hellberg and Hopkins (2006).

In New Zealand, *B. exitiosa* is considered endemic and has caused significant epidemics in wild native flat oyster O. chilensis, particularly since the mid‐1980s (Cranfield et al., 2005; Doonan et al., 1994; Michael et al., 2016). *B. ostreae*, however, was first detected fairly recently (Lane et al., 2016) and this detection (and subsequent events) generated surveillance programs and research initiatives dedicated to modeling the spread risk, and to informing management strategies.

Modeling should be informed by existing literature and experimental results. However, the majority of the experimental work and published research on Bonamia species examines either the epidemiology and pathology of *B. exitiosa* affecting *O. chilensis* in New Zealand (Cranfield et al., 2005; Diggles & Hine, 2002; Hine, 2002, 1991; Michael et al., 2016) (i.e., the wrong species of parasite), or *B. ostreae* affecting *Ostrea edulis* (*O. edulis*) in Europe (Engelsma et al., 2014) (i.e., the wrong host oyster species).

As a consequence, the initial conceptual model built for this project (by researchers at Cawthron Institute) was informed by a body of literature, which contains very little information about *B. ostreae* affecting *O. chilensis*. Nevertheless it is crucial to collate and summarize all available information that may inform the modeling. The processes summarized in the following section are the building blocks of the modeling and was provided as a background document to all experts involved in the elicitation process.

### 3.2 Modeled Processes

**FIGURE A1:**
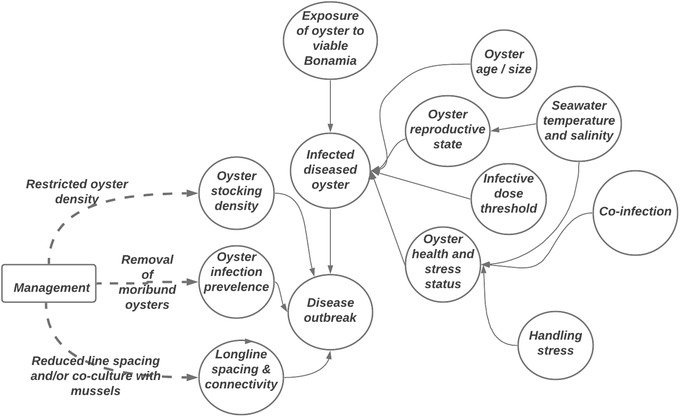
The conceptual local‐scale model for a disease outbreak within a two‐dimensional wild bed or a three‐dimensional farm space. The arcs and the nodes are not meant to represent variables and causal relationships, but factors connected tentatively

We considered the starting conceptual model to be the local‐scale model for a disease outbreak within a two‐dimensional wild oyster bed or a three‐dimensional farm space (where oysters are grown above the seabed). In the initial schematic representation of the problem, developed by the problem owners, the endpoint was an “outbreak”(see Figure A1 from the Appendix^7^). However, prior to an outbreak, the condition could present as a persistent low‐level infection in individuals, or with only low prevalence and low levels of mortality in the population. This may remain undetected for years before the infection in a farm, or a wild bed, becomes an outbreak. Therefore, it is desirable to model both (i) the risk of an actual outbreak and (ii) the disease risk from infected populations in the absence of an observed disease outbreak. To achieve these goals, the model should predict the low‐level release of parasites from infected but apparently healthy oysters, as well as from heavily infected and dying oysters. To accommodate this range, model outputs needed to include quantitative estimates of both the number of individuals infected in a population, and of the number of *B. ostreae* parasites from infected or dying hosts released in a given time frame. The likelihood of infection in a population depends (directly or indirectly) on the characteristics of that population and the susceptibility of the uninfected population, under specific environmental conditions (water salinity, water current speed and temperature).

A host population can be characterized by the size distribution, the age distribution, the reproductive state distribution, and the health status of its individuals. The latter is in turn influenced by the presence of stresses (handling stresses or coinfections) and environmental factors, for example, Bradley et al. (2020). All these factors were considered and discussed through the initial stage of the modeling. The following subsections summarize the factors (and their interdependencies) that became part of the model; these will be highlighted in the text.

#### 3.2.1 Infection Intensity and Number of Released Parasites

The two desired output variables are closely related to the infective and lethal doses^8^ in a particular animal or population. Even though the infective dose threshold is present in the first schematic representation of the problem, and its relationship with the host population characteristics was discussed, this relationship is currently unknown for *O. chilensis* infected by *B. ostreae*. The model needs to be based on assumptions informed by the available published research on the infective and lethal doses of *B. ostreae* affecting *O. edulis*. This (published) research has demonstrated that a threshold dose is usually required to achieve infection of an animal. As an indication of this threshold, the ID50 (to cause detectable infection after four months) was found to be 80,000 parasites per oyster in three‐year‐old *O. edulis* (Hervio et al., 1995). Hervio et al. (1995) found that a single parasite directly injected into the digestive gland only caused an infection in 1/150 oysters. Actual levels of mortality from bonamiosis, and levels of infection intensity in an individual are difficult to predict despite a large amount of research on the topic (Arzul et al., 2011; Caceres‐Martinez et al., 1995; Culloty et al., 2004, 1999; Culloty & Mulcahy, 1996; Hervio et al., 1995; Lallias et al., 2008; Lynch et al., 2005, 2014; Martin et al., 1993; Mialhe et al., 1988; Montes, 1991; Montes et al., 2003; Naciri‐Graven et al., 1998; Poder et al., 1982; Tige & Grizel, 1984). These predictions vary depending on a range of environmental factors, and intrinsic host factors (e.g., age, size, health status, selectively bred for bonamiosis resilience status). *Infection intensity* and its relationship with host factors became part of the conceptual model.

#### 3.2.2 Time to Death

When an infection becomes lethal, upon death, a large load of parasites is released into the water column from the dying and decaying oyster. Oysters will decay very quickly once dead, and up to 443,000,000 parasites^9^ have been isolated from a single heavily infected adult *O. edulis* (Lallias et al., 2008). However, mortality at the population level and individual time to death vary hugely, depending on environmental and host factors (discussed further in Section 3.2.4).*Time to death*, even though not in the original schematic representation, was added to the model as one of the factors, which directly influence *the number of parasites released per month* (together with *the infection prevalence* and the *size of the host population*).

#### 3.2.3 Parasite Survival and Seasonality

Seasonality (mainly driven by temperature) plays an important role in the host behavior (and this is further discussed in Section 3.2.4). Moreover, *temperature* influences the survival of the parasite. The *B. ostreae* parasites exist as tiny microcells (≈2μm in diameter) and their survival depends mainly on temperature. Arzul et al. (2009) found that parasites may survive at least 48 hours, with significantly higher survival at 15 ${}^{\circ }$C compared to 25 ${}^{\circ }$C. Because the reported experiments were only run for 48 hours, it remains unknown for how long parasites might exist outside the host under optimal conditions. Feng et al. (2013) confirmed survival of the parasite after one month at 4 ${}^{\circ }$C in a tissue homogenate, but at very low temperatures ($\approx 0{\;}^{\circ }$C) the parasite appears to be killed or at least not proliferate (Madsen et al., 2013). These experiments informed the modeling of *viable parasite abundance* and the seasonality aspect of the modeling process.

#### 3.2.4 Host Susceptibility and Seasonality

All ages/sizes of oyster appear to be susceptible to Bonamia infection, as *B. ostreae* has been detected in *O. edulis* larvae (Arzul et al., 2011), and *O. edulis* spat can demonstrate a high prevalence of infection of *B. ostreae* (Lynch et al., 2005). However, larger oysters often appear to have the highest prevalence of infection and mortality levels, particularly postspawning (Caceres‐Martinez et al., 1995; Culloty & Mulcahy, 1996; Hine, 1991, 1996). Seasonal peaks in prevalence have been observed particularly in the spring (Engelsma et al., 2010) or in early autumn following spawning (Caceres‐Martinez et al., 1995; Carnegie et al., 2008; Culloty & Mulcahy, 1996; Hine, 1991, 1996). The presence of stresses (*handling stresses* or coinfections) and environmental factors are also implicated in the clinical manifestation of *B. exitiosa* in farmed oysters in Australia (Bradley et al., 2020) and similar observations had been anecdotally noted for *B. ostreae* in New Zealand. In *O. chilensis* in New Zealand, variation in spawning timing and prevalence shows a latitudinal trend with temperature from north to south (Brown et al., 2010; Jeffs, 1998; Jeffs et al., 1997, 2002; Stock, 2000).

#### 3.2.5 Transmission Processes

Transmission of both *B. ostreae* and *B. exitiosa* is direct host to host, without the need of an intermediate host. The transmission distance depends on hydrodynamic processes, which will be informed by a separate hydrodynamic model. Nevertheless, there is some published literature that indicates that natural spread is limited and slow (Howard, 1995; Laing et al., 2014). However, there may be unknown intermediate hosts (Lynch et al., 2010), or other species may act as mechanical vectors and transport parasites.

#### 3.2.6 Host Density and Cocultures

Mortality on farms in New Zealand may have been positively correlated to the stocking densities on those farms (A. Elliot and B. Hearn, personal communication). In addition, it was observed that oysters on lines hanging very close to other lines, which had very high levels of mortality, could have no detectable Bonamia infection (B. Hearn, personal communication), indicating that local hydrodynamic factors may be highly important as well as stocking *density*. This is consistent with older research on the *B. exitiosa* prevalence in the wild beds fished in Foveaux Strait (Doonan et al., 1999; Hine, 1996).

It has been anecdotally observed in New Zealand that mortality can be lower in farmed oysters that are heavily covered with fouling organisms (B. Hearn, personal communication). It is also hypothesized that mortality may be reduced by coculture with mussels or another filter feeding bivalve that is resistant to the parasite (e.g., C. gigas). There is limited published evidence to support this hypothesis, for example, Ben‐Horin et al. (2018), but given that coculture with mussels was often the culture technique employed in New Zealand prior to the arrival of *B. ostreae*, it certainly warrants further investigation as a potential mitigation tool. Even though discussed with the group of experts, cocultures were not modeled at this stage.

### 3.3 Local Bonamia BN

**FIGURE 2:**
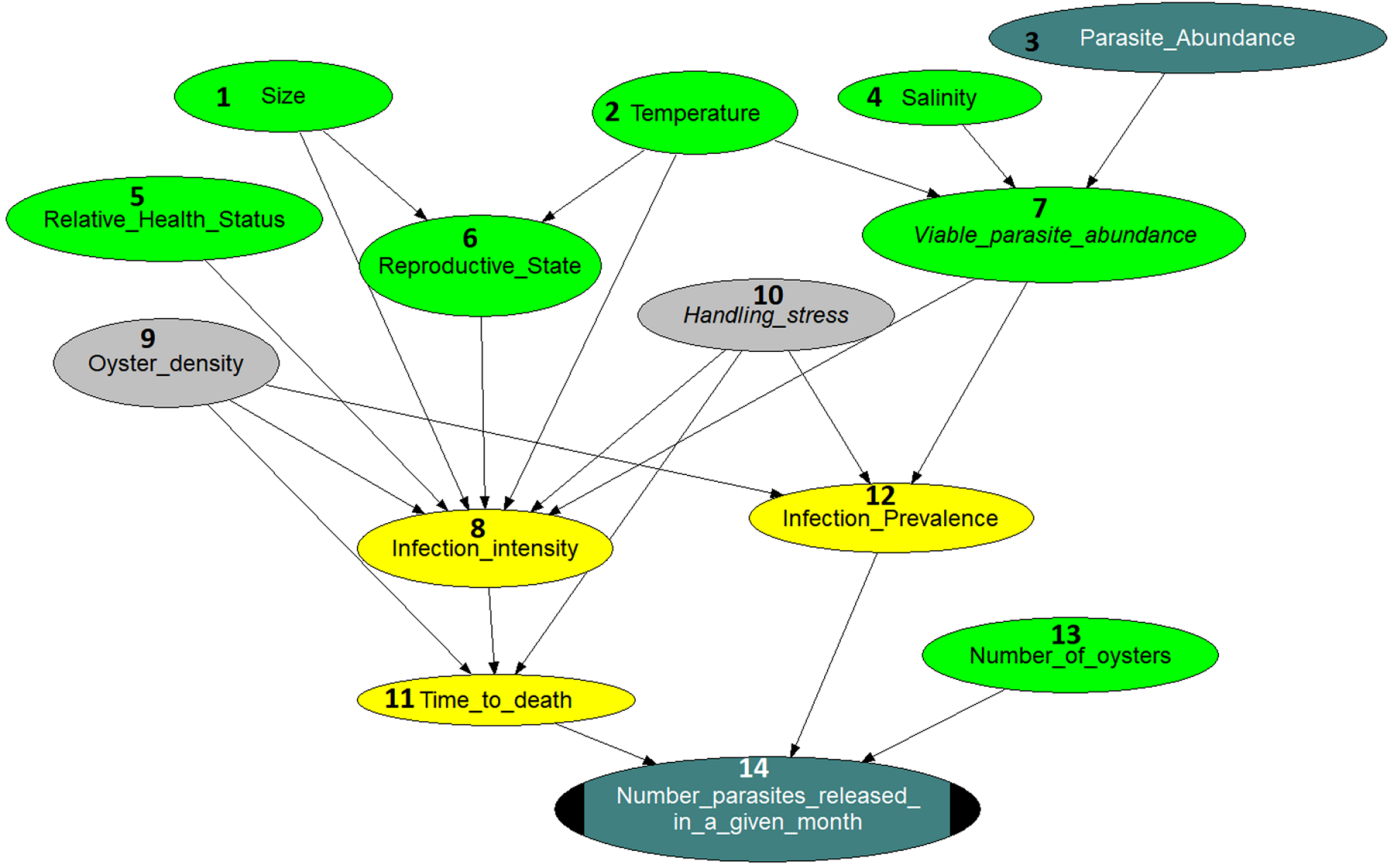
Local Bonamia BN model representing the behavior of an oyster population located in the Tory Channel. The light green nodes are quantified with data, the yellow ones are quantified with experts. The dark green nodes represent functional variables and the gray nodes represent constants

**FIGURE A2:**
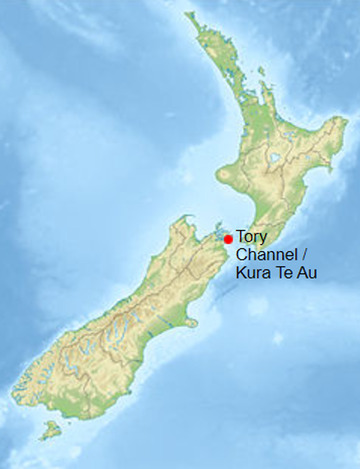
Tory Channel

**FIGURE A3:**
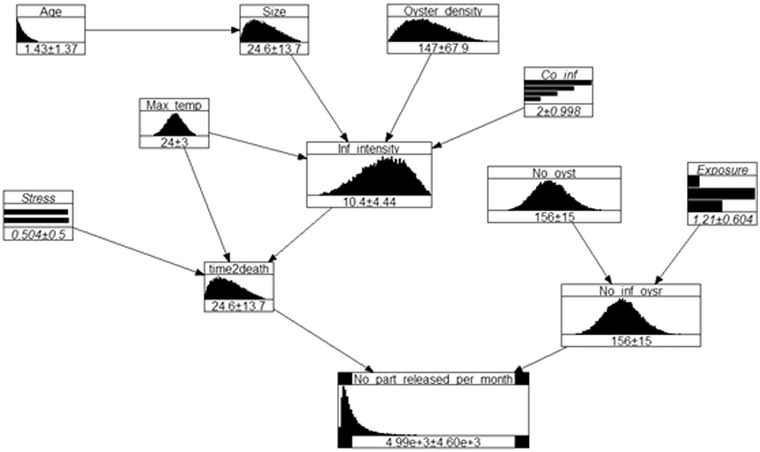
Initial local *Bonamia* BN model representing the behavior of an oyster population located in the Tory Channel

The intention is to model the behavior of a single population of oysters of varying sizes and ages located in the Tory Channel, Marlborough (see Figure A2 from Appendix). This location has constant oceanic salinity, sea water temperature typically varying between 12 ${}^{\circ }$C and 17 ${}^{\circ }$C over a year, and strong tidal flow with very little freshwater influence. The time step of the model is a month, with simulations starting in March. Based on the conceptual model, the elicitation team built an initial, draft BN structure (shown in Figure A3 from the Appendix) that was the basis for the final model shown in Figure 2. Each node should represent a factor/variable and, ideally it should be described by a probability distribution. However, to keep the model parsimonious, some of the nodes (the gray ones) were kept constant. The nodes in Figure 2 are color coded to represent quantification type and sources (e.g., data or expert judgments, fixed value, or deterministic relationship) and have numbers associated with them for easier reference. Arcs (arrows) are associated with correlations, which are meant to define the strength of dependencies between connected nodes.

The process of building, refining, and quantifying the model was described in detail in Section 2.2. The literature used to further inform modeling for this particular case study is detailed in Section 3.2. Both the literature and how it informed the modeling, and the model structure were scrutinized by subject matter experts, and guided by the probabilistic modeling constraints of BNs. As noted earlier, a BN model is based on factorizing a multidimensional interconnected set of factors/variables into subsets, by distinguishing between direct and indirect influences (represented by arcs). For example, in the model from Figure 2, the variable 11 (Time to death) of a random infected oyster is directly influenced only by its parents (8 [Infection intensity], 9 [Oyster density], and 10 [Handling stress]), even though it clearly depends on other variables modeled. The reason for this is that the latter dependence is mediated by the set of parents (which represent the direct dependencies). This type of factorization translates into a lower quantification burden, but it can be seen as a modeling restriction, namely, the set of one child's parents should not be too large.

From the same considerations, not all nodes represented random variables. Some were kept constant (the gray nodes in Figure 2) for simplicity, but also because their interdependence with the rest of the variables was based on hypotheses that were not supported by observational data, but were instead conjectured by experts.

To fully incorporate such constants, and other potential factors that were not included as nodes in the conceptual modeling stages, but are important conditioning factors, we proposed distinct scenarios that cover the possible different situations. These scenarios are defined by a set of conditions that describe the information and speculations about the effects of the oyster population density, the external stresses, the proximity to another population of filter‐feeding organisms. The combination of these factors also act as proxies for the type of modeled population (farmed or wild). These scenarios were constructed and refined with the expert group and they were crucial for keeping the model parsimonious.

#### 3.3.1 Scenarios

To cover the variability of density, two types of populations were chosen, a high‐ and low‐density population: Oysters are considered to be spread out evenly at either 5 cm, or at 50 cm interindividual spacing. The effect on the spread of parasites of having another population of bivalves nearby was incorporated in a scenario that proposes that the oyster population is adjacent to a mussel farm, and assumes that mussels are not able to act as hosts and multiply the parasite in the environment. Bonamia parasites are negatively buoyant, which means that a proportion of them will sink, however, it was assumed that all parasites come in contact with a “curtain” of mussels^10^ (based on long‐line type farming used in mussel farms from New Zealand). We assumed the mussels nearby were both upstream and downstream of the oysters due to the currents and tides.

“Handling stress,” a factor that is thought to influence the infection intensity of a potentially diseased oyster, represents stress from physical manipulation such as, for example, grading on a farm, or dredging in a wild bed (see Figure A1). For simplicity, degrees of such manipulations were not considered, but rather handling stress was considered to either occur or not, and these situations were captured by different scenarios. The four scenarios were as follows:

Scenario 1
The average interoyster distance to the nearest neighbor is 50 cm; the oysters are handled (e.g., graded, defouled, dredged, or otherwise disturbed) every three months.
Scenario 2
The average interoyster distance to the nearest neighbor is 50 cm; oysters are never handled.
Scenario 3
The average interoyster distance to the nearest neighbor is 5 cm; oysters are never handled.
Scenario 4
The average interoyster distance to the nearest neighbor is 50 cm and the oysters are never handled. However in this scenario, unlike in Scenario 2, the oyster population is situated immediately next to a 1 ha. mussel farm of 40 million mussels grown on long lines as per normal mussel farming practices in New Zealand (e.g., 160 mussels per linear meter of rope, equivalent to 40 mussels per m${}^{3}$ of water space).

Any of the scenarios can be thought of as corresponding to either a wild oyster bed (that has high or low density, and it is either dredged or not) or a farm (with high or low stocking density, and with or without oyster grading). The wild oyster bed or farm may be located next to a mussel farm (Scenario 4). Scenarios 1, 2, and 4 can be thought of as corresponding to increasingly better conditions, when the density is held constant: one, where oysters are handled (Scenario 1), another where oysters are never handled (Scenario 2), and the last, where oysters are never handled and are situated in the proximity of a mussel farm. Scenario 3 is a high‐density scenario without handling stress; its comparison with Scenario 2 isolates the influence of oyster density.

#### 3.3.2 Nodes, Arcs, and Their Quantification

The model output is a single point of parasite release in a given month (node 14). The output of the model is used as an input (node 3) of the same model, to advance the calculations for the next month (the starting month is March). In this way, the dynamic/temporal nature of this physical system is mimicked and the seasonal variations of the processes are accounted for. Some of the variables' distributions change every month (e.g., node 2, node 4, and all variables directly or indirectly influenced by node 3), whereas others retain the uninformative, generic distributions chosen initially. The latter choices are made for simplicity, in accordance with the expert group and are identified as limitations later in the article.

What follows is a description of the nodes, their interconnections, and the sources of quantification used for their distributions or the rank correlations.

- 1 (Size) is the size distribution of oysters in the population measured in mm diameter. This distribution may be informed by data, but in the absence of data, a uniform distribution from 30 mm to 100 mm is used.
- 2 (Temperature) is the sea‐surface temperature occurring over a given month at the model site and it is quantified using data collected from Tory Channel.
- 4 (Salinity) is the range of minimum to maximum values for salinity occurring over a given month at the model site and it is quantified as a probability distribution informed by data collected from Tory Channel.^11^2 (Temperature) and 4 (Salinity) are used to calculate mortality of the Bonamia parasites over time using a submodel fitted on the experimental data from Arzul et al. (2009). While not explicitly included, mortality information contributes to the calculation of 7 (Viable parasite abundance) (described below).
- 3 (Parasite abundance) is the number of Bonamia parasites in the water space occupied by the population. This can be determined by the output of the model at a previous time step (see 14 [Number of parasites released in a given month]). For simplicity, we ignore the space dimension, which will be incorporated once this model is combined with hydrodynamic modeling. For the purpose of this model, this variable is initiated with a prior uniform distribution on $\left(10^{4},10^{9}\right)$ and sequentially updated with every (monthly) model run.
- 5 (Relative health status) is a descriptor that attempts to integrate an entire range of factors (except temperature and salinity) that could affect host susceptibility and resilience to both initial infection and parasite replication. This would include factors such as coinfections, food availability, condition index. This simplification seems artificial but was agreed upon by the group of experts. The relative health of the oysters is modeled by a normal distribution with most of the mass between −1 and +1, where 0 represents an average healthy oyster, −1 corresponds to the worst health condition relative to the average, and +1 corresponds to the the best health condition relative to the average.^12^ The relative health status influences (is linked to) the infection intensity. This variable is modeled as a Normal distribution with mean zero and 0.25 standard deviation. It may be argued that the health status also influences the infection prevalence (node 12 in Figure 2) due to potential effects on relative susceptibility. This influence was discussed, but not modeled as a direct link between nodes 5 and 12 (to preserve model simplicity). However, even in this simplified form, the contribution of the health status to the final output of the model is captured indirectly.
- The sixth (Reproductive state) variable is defined as the proportion of individuals in the population who are resorbing gonadal tissue^13^ in any given month. This node was informed by data on farmed oysters from Marlborough,^14^ and from wild populations (Brown et al., 2010). The resorption process has been estimated based on observing the number of animals brooding in each month and assuming that resorption occurs in the month following brooding. Based on these data, the reproductive state can be represented as the probability of a random individual (from the given population) to be resorbing. 6 (Reproductive state) is influenced by both size and temperature, and these relationships, described in terms of rank correlations were informed by data from Brown et al. (2010) and Brown (2011).
- The seventh (Viable parasite abundance) variable is the number of viable infective Bonamia parasites in the water column in the space occupied by the population. The viable parasite abundance is calculated as a function of mortality (modeled based on temperature and salinity) and parasite abundance according to relationships derived from experiments on survival of *B. ostreae* in seawater from Arzul et al. (2009). This submodel is dealt with externally, and the resulting outputs are used in the NPBN. The viable parasite abundance was discretized in three levels: low, moderate, and high^15^ when used as a conditioning variable for quantifying the infection prevalence using expert judgment. Discretizing was considered necessary in order to alleviate the elicitation burden.
- The eighth (Infection intensity) is the actual number of parasites in a random infected individual oyster. It is represented by the probability distribution for the population. It may be influenced by the size, health status, reproductive state, and the abundance of viable parasites. It is also influenced by the temperature, host density, and potential stresses. The range and best estimate of infection intensity across a given year were elicited from experts. So were its correlation with the relative health status, the size, the reproductive state, the temperature, and the viable parasite abundance.
- 9 (Oyster density) is defined as the shortest distance to the nearest oyster neighbor, rather than the number of individuals per m${}^{3}$, in order to make the measure applicable to any type of oyster population, for example, a three‐dimensional long‐line farm or a flatter benthic oyster bed. Density was fixed by the scenarios. As a consequence, nothing was elicited for this node (neither a distribution, nor correlations corresponding to outgoing arcs).
- 10 (Handling stress) is defined as the presence or absence of stress due to grading or cleaning biofouling off farmed oysters, or dredging in a wild bed. This is fixed by the scenarios as present or absent. As a consequence, nothing was elicited for this node (neither a distribution, nor correlations corresponding to outgoing arcs).
- 11 (Time to death) is the estimated time in months until a random infected individual will die. This may be influenced by the infection intensity, the oyster density, and the presence of handling stress. This variable's distribution was elicited from experts along with its correlation with the infection intensity.
- 12 (Infection prevalence) is the proportion (%) of individuals in the population that are infected with any amount of Bonamia. This may be influenced by the abundance of viable parasites in the water, the presence of handling stress, and the oyster density. The infection prevalence in the given population was elicited from experts conditional on three levels of the viable parasite abundance and the other scenario‐dependent fixed parameters. Note that in this case a conditional distribution was elicited to represent dependence (rather than a correlation). This decision was taken by the experts who considered thinking in terms of correlation unintuitive in this case.
- The 13th (Number of oysters) is the total number of oysters in the population and it was represented as a uniform distribution on (80,000, 120,000). The mean of this distribution is 100,000 as initially considered.
- The 14th (Number of parasites released in a given month) is the total number of Bonamia parasites released from the whole population over the course of a given month. This is a mathematical function based on the time to death of infected oysters, the infection prevalence in the population, and the total number of oysters in the population. The infection prevalence multiplied by the total number of oysters results in the number of infected oysters, which divided by the time to death (in months) and multiplied by the number of parasites released by an infected oyster upon death^16^ results number of parasites released in a given month.

The arcs in the model from Figure 2 represent potential dependencies. Most of these dependencies are represented as rank correlations in the model. The exceptions are the arcs directly influencing 14 (Number of parasites released in a given month) and those influencing 7 (Viable parasite abundance). These nodes have a functional relationship (representing physical models) with their parents, so the corresponding five arcs represent that relationship rather than probabilistic dependencies. Six of the correlations were elicited from experts, and three were estimated from data (as identified above). One relationship is elicited as a conditional distribution of the child (12 [Infection prevalence]) given its discretized parent (7 [Viable parasite abundance]) and the rank correlation between parent and child is calculated from this information. Six of the arcs disappear when two of the variables are considered constants rather than random variables and dealt with through the formulated scenarios.

The best possible aggregation of elicited values (discussed in Section 2.2) together with the available data, generated everything that was needed for the quantification of 48 models: 12 models (one per month) × 4 scenarios. These models were implemented in the https://lighttwist‐software.com/uninet/UNINET software for NPBNs. Each model outputs the number of parasites released per month. Little variation was observed between scenarios, suggesting that the density, the handling stress, and proximity to a mussel population were not thought to add too much information additional to the other modeled variables, or to the seasonal effects. This however should only be thought of as a signal, rather than a definitive conclusion, given all the simplifications and assumptions of the models.

**FIGURE 3:**
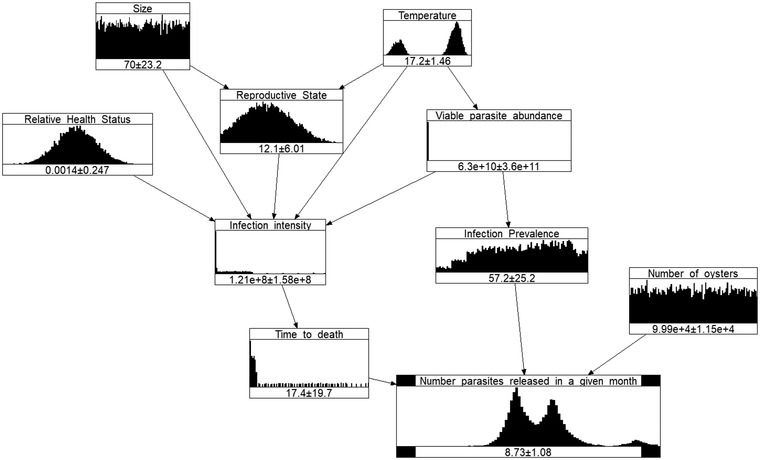
Summer Bonamia BN model for Scenario 3, as represented by an “average” January

Discussing the distributions of the variables is out of the scope of this article, however, a few observations are in order. Figure 3 shows the distributions of the variables under Scenario 3, for the summer (as represented by the month January).

When running the models, the constant values for 9 (Oyster density) and 10 (Handling stress) are not represented as nodes anymore (but their values are still used in the calculations for each scenario). Even though useful in the visualization of the original model, nonprobabilistic nodes are not represented in the NPBN, since all the nodes of BNs represent random variables.

The 3 (Parasite abundance) and the 4 (Salinity) nodes, even though probabilistic, are absorbed into the 7 (Viable parasite abundance) node (when running the external submodel), since this is their only child (i.e., they do not influence any other random variable from the model). When nodes disappear so do the arcs originating from them, as their influence becomes implicit in the calculations. The values of the output variable are presented on a logarithmic scale for better visibility. The numbers at the bottom of the nodes represent means and standard deviations.

The unusual shape of the 8 (Infection intensity) and 11 (Time to death) variables is caused by many factors. The most important is that the experts gave only three percentiles, and only those are used to build a nonparametric distribution (where the mass is simply spread equally between the three percentiles). Furthermore, the experts expressed large uncertainty about the infection intensity, and that is reflected in the fact that its values spread three orders of magnitude. However, lower values were considered more probable than higher values, resulting in a long right tail of the distribution. The 11 (Time to death) distribution is somewhat similar in the sense that its support ranges from a couple of months to four years, and it has a long right tail.

The other variable elicited from experts,12 (Infection prevalence), was elicited as a conditional distribution, which was then marginalized and sampled from, producing a smoother shape.

The uniform distributions for 1 (Size) and 13 (Number of oysters) represent uninformative priors, to be updated whenever more information becomes available.

**FIGURE 4:**
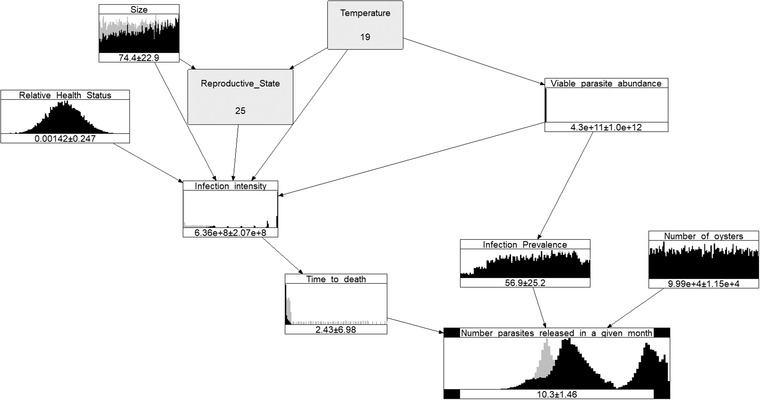
Summer Bonamia BN model for Scenario 3, as represented by a hot January (Temperature $=19^{\circ }$C) with a high value for the oysters' Reproductive state (equal to 25% of the population resorbing)

**FIGURE 5:**
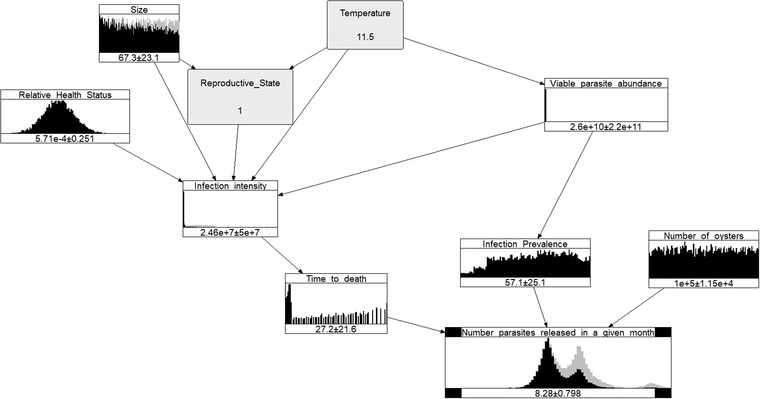
Winter Bonamia BN model for Scenario 3, as represented by a cold July (2 [Temperature] $=11.5^{\circ }$C) with a low value for the oysters' 6 [Reproductive state] (equal to 1% of the population resorbing)

To illustrate seasonal difference we look at January (representing summer, Figure 4) and July (representing winter, Figure 5) under the conditions of Scenario 3. The parasites release in summer is far greater than in winter and the differences are more visible when we conditionalize the model on a very hot summer and a very cold winter.

Figure 4 shows the conditional distributions of variables given Temperature $=19^{\circ }$C and Reproductive state =25%. The gray distributions correspond to the unconditional ones and are provided for comparison. The mean logarithm of the number of released parasites increased from 8.73 to 10.3.

When we conditionalize on a cold winter and a low reproductive state (2 [Temperature] $=11.5^{\circ }$C and 6 [Reproductive state] =1%), the mean logarithm of the number of released parasites drops to 8.28 (see Figure 5).

We reiterate that these numbers should be treated with caution and scrutinized further in the next stage of the modeling.

## 4 DISCUSSION

It is worth reiterating that this research constitutes the first building block of a much larger risk model. It acts as a proof of principle for the methodology and it explores a realistic small‐scale risk modeling component that can easily be improved and extended.

The two main strengths of the proposed process are: (1) its participatory, iterative, and structured approach; and (2) the probabilistic tools used in the modeling.

The first one proved essential in building a consensus model and eliciting expert data. The cooperation and collaboration of a large number of international subject matter experts, analysts, and stakeholders were not only highly informative from a modeling perspective, but had also beneficial effects related to the credibility of the modeling and to an increased overall understanding of the problem.

The second strength, namely, the use of NPBNs, guaranteed a framework, which lends itself well to flexible probabilistic modeling in data‐sparse environments. Augmenting an NPBN with nodes and arcs can happen naturally, whenever data become available, or experts are comfortable to expand the model. Prior distributions (used for convenience) can be simply replaced by more informative ones, and nodes can be replaced (or informed) by submodels when more complexity is needed.

However, we advise caution when interpreting the numbers generated by this research, due to the acknowledged limitations of the study. These limitations are either related to modeling choices and assumptions, or to methodological/theoretical shortcuts. For example, the intention was to model and estimate parasite release from live and dead oysters; however, we assumed that the contribution from the live oysters is negligible when compared to the number of parasites released by dead ones. When data become available we can easily model this contribution if it proves significant.

The relative health status was modeled as a generic variable, whereas in a more detailed model it could be the result of a more complicated stress submodel (incorporating coinfections, food availability, condition index, etc., and potentially the handling stress).

We essentially constructed a model as a two‐dimensional domain by ignoring the space occupied by the population. For integrating the model with a hydrodynamic model, this space is essential. Parasite abundance will inform the concentration of parasites in the water column, which will then interact with the depth and hydrodynamics of a given site. Together with the density of the parasites, these will determine the dilution of the parasites and their maintenance in the water column.

A more nuanced population may be considered (as opposed to just wild and farmed) for which the origin of the oysters (e.g., hatchery, wild, selectively bred) is better specified. The current model is based on a naive population and therefore may significantly overestimate the infection intensity and prevalence in a more stable and established host–parasite system, as it has been observed for *B. ostreae* in *O. edulis* in Europe after its initial introduction from North America (Culloty et al., 2001, 2004).

From a methodological viewpoint, we combined different methods of eliciting and representing dependence: We elicited rank correlations and derived (constant) conditional ranks to parameterize the BN, we elicited conditional distributions (based on a discretized version of one of the variables) and derived correlations, which were later combined with the elicited correlations. The final correlation matrix does not comply with all of the aggregated correlations. All these transformations may have induced biases, which are impossible to correct for at the moment. Moreover, the data sets used to complement the expert judgments were fairly limited.

Despite these limitations, none of which is insurmountable, our exploration of the NPBN suggests that the model produces plausible outcomes that are generally consistent with the available data, the described seasonal relationships, and the expert assessments. While future improvements of the modeled case study are possible, the model presented here provides a useful initial framework (consistent with the best available information) for exploring management decisions in this region.

## Supporting information

Supporting Information

## Footnotes

1 There are hundreds of heuristics that people use when reasoning under uncertainty. These lead to biases that cannot be eliminated but can be mitigated against. Most elicitation protocols try to mitigate against anchoring, availability, overconfidence, and group think.

2 In practice this requires calibration questions, which ideally are questions for which the true answers will soon become available.

3 These numbers do not take into account the experts from the elicitation team, needed for formulating the elicitation questions and advising on the question format.

4 One of the experts from the initial 20 dropped out, another was recruited, and two out of the 20 experts were part of the elicitation team rather than the expert group.

5 The questions are available as Supporting Information.

6 For the elicited correlations, only the best estimates were used since we were not interested in building distributions of correlations.

7 Figure A1 is placed in the Appendix to avoid potential confusion due to the similarities between the two graphical representations of (1) the initial mind map and (2) the probabilistic graphical model. In the latter, nodes represent random variables and arcs represent either cause–effect or correlational relationships. In contrast, the former only served as a conversation starter between the problem owner and the analysts, with no formal meaning of nodes and arcs.

8 The infective dose (ID50) is the dose where half of the population become infected, and the lethal dose (LD50) is the dose at which half of the animals will die (within a given time frame).

9 Based on this number we have used an approximation of 500 mill parasites as a multiplication factor when calculating the number of parasites released per month upon the death of individuals.

10 The model does not consider any anthropogenic spread (e.g., from boats or equipment) so it assumes that there is no farm, just a curtain of mussel.

11 The data were collected by a profiling conductivity–temperature–depth (CTD) instrument for Tory Channel from which we have summarized surface seasonal temperature and salinity data for the model simulations.

12 The experts specified the shape and the approximate limits of the distribution and the modelers fitted a normal distribution with zero mean and 0.3 standard deviation.

13 As per the definition of Hine (1991) and Jeffs and Hickman (2000).

14 The oysters were assessed via histology on a monthly basis and resorption was observed directly, over three different years

15 Low corresponds to 1,000 or less parasites present in the surrounding water which may come in contact with the oysters, moderate corresponds to values between 1000 and 100,000 parasites, and high corresponds to values larger than 100,000 parasites. These thresholds are informed by Hervio et al. (1995)

16 This number was fixed to 500 millions as informed by the literature in Section 3.2.
